# Supplementary material for: Experimental Examination of Solubility and Lipophilicity as Pharmaceutically Relevant Points of Novel Bioactive Hybrid Compounds
Source: Molecules. 2022 Oct 1;27(19):6504. doi: 10.3390/molecules27196504 (PMC9573696; doi:10.3390/molecules27196504)
Supplement: Supplementary file 1 [file molecules-27-06504-s001.zip › molecules-1935370-supplementary.pdf]

Supplementary Material
for
Experimental Examination of Solubility and Lipophilicity as Pharmaceutically Relevant Points of Novel Bioactive Hybrid Compounds

Angelica Sharapova, Marina V. Ol'khovich, Svetlana Blokhina and German Perlovich*

Institute of Solution Chemistry, Russian Academy of Sciences,

1 Akademicheskaya Street, 153045, Ivanovo, Russia

Content:

Table S1. Kinetic dependencies of solubility of compounds studied 2

Table S2. Group contribution parameters and associated molar volumes of compounds studied. 3

Table S3. Experimental (x_{exp}) and correlated (x_{cal}) mole fractions of compounds studied (amorphous state) solubility in the solvents studied at different temperatures and pressure p = 0.1 MPa. 3

Table S4. Parameters of modified Apelblat and van't Hoff equations for of compounds studied (amorphous state) in the selected solvents. 4

Table S5. Coefficients of empirical equation $\ln x = A - B/(1/T\text{-}1/T_{\text{hm}})$. 5

Figure S3. Relationship of the enthalpy and entropic terms of the dissolution Gibbs energy for hybrid compounds I – III.

Figure S1. PXRD patterns of amorphous compounds I – III. 6

Figure S2. DSC curves of compounds I (A), II (B) and III (C). 7

Table S6. Density of the investigated solvents at different temperatures and pressure p = 0.1 MPa 8

Table S1. Kinetic dependencies of solubility of compounds studied I - III in buffer solutions.

Buffer pH 2.0		Buffer pH 7.4	
t/min	S/mol·L^{-1}	t/min	S/mol·L^{-1}
		**I**	
0	0	0	0
30	$7.11 \cdot 10^{-4}$	30	$5.86 \cdot 10^{-6}$
90	$1.24 \cdot 10^{-3}$	60	$8.68 \cdot 10^{-5}$
150	$1.61 \cdot 10^{-3}$	90	$1.13 \cdot 10^{-4}$
384	$1.93 \cdot 10^{-3}$	186	$1.22 \cdot 10^{-4}$
622	$2.01 \cdot 10^{-3}$	321	$1.23 \cdot 10^{-4}$
1440	$2.13 \cdot 10^{-3}$	1400	$1.24 \cdot 10^{-3}$
2880	$1.98 \cdot 10^{-3}$	1700	$1.24 \cdot 10^{-3}$
3180	$1.98 \cdot 10^{-3}$	2880	$1.24 \cdot 10^{-3}$
		3180	$1.24 \cdot 10^{-3}$
		**II**	
0	0	0	0
30	$6.10 \cdot 10^{-4}$	30	$9.153 \cdot 10^{-6}$
90	$7.10 \cdot 10^{-4}$	60	$9.611 \cdot 10^{-5}$
150	$9.21 \cdot 10^{-4}$	90	$2.002 \cdot 10^{-4}$
210	$9.98 \cdot 10^{-4}$	186	$2.971 \cdot 10^{-4}$
384	$1.11 \cdot 10^{-3}$	321	$3.383 \cdot 10^{-4}$
622	$1.18 \cdot 10^{-3}$	1400	$3.707 \cdot 10^{-4}$
1440	$1.31 \cdot 10^{-3}$	2880	$3.192 \cdot 10^{-4}$
2880	$1.24 \cdot 10^{-3}$	4320	$3.192 \cdot 10^{-4}$
4320	$1.23 \cdot 10^{-3}$		
		**III**	
0	0	0	0
39	$3.43 \cdot 10^{-4}$	20	$1.65 \cdot 10^{-5}$
65	$4.20 \cdot 10^{-4}$	50	$3.11 \cdot 10^{-5}$
214	$4.80 \cdot 10^{-4}$	113	$4.71 \cdot 10^{-5}$
280	$4.89 \cdot 10^{-4}$	180	$6.14 \cdot 10^{-5}$
385	$5.07 \cdot 10^{-4}$	280	$6.27 \cdot 10^{-5}$
1422	$6.09 \cdot 10^{-4}$	335	$6.34 \cdot 10^{-5}$
2682	$6.76 \cdot 10^{-4}$	1350	$6.65 \cdot 10^{-5}$
2880	$6.78 \cdot 10^{-4}$	2690	$6.73 \cdot 10^{-5}$
		2880	$6.73 \cdot 10^{-5}$

Table S2. Group contribution parameters and associated molar volumes of compounds studied.

Individual functional group	Frequency	F_{di}, $(J/cm^3)^{0.5}\cdot mol^{-1}$	F_{pi}, $(J/cm^3)^{0.5}\cdot mol^{-1}$	E_{hi}, J/mol	V_i, cm^3/mol
		**I**			
-F	2	102.0	493.9	6544.3	18.0
=CH-	10	255.0	38	0	13.5
$-CH_2-$	9	234.6	0	0	16.1
>C<	1	-214.2	0	0	-19.2
-OH	1	76.5	1225.0	6060.0	10.0
-CO-	1	105	600.0	9500.0	10.8
$-CH_3$	1	336.6	0	0	33.5
-S-	2	815.9	196.0	297.5	12.0
=C<	7	56.7	2.00	0	5.5
-N=	3	380	100	250	5.0
>N-	5	30	150.0	750.0	-9
Ring closure 5 or more atoms	4	142.8	0	0	16
Phenylene	2	1173.0	63.7	40.4	52.4
Double bond	12	15.0	14.3	83.5	2.2
		**II**			
-F	3	102.0	493.9	6544.3	18.0
=CH-	10	255.0	38	0	13.5
$-CH_2-$	9	234.6	0	0	16.1
>C<	1	-214.2	0	0	-19.2
-OH	1	76.5	1225.0	6060.0	10.0
-CO-	1	105	600.0	9500.0	10.8
-S-	2	815.9	196.0	297.5	12.0
=C<	7	56.7	2.00	0	5.5
-N=	3	380	100	250	5.0
>N-	5	30	150.0	750.0	-9
Ring closure 5 or more atoms	4	142.8	0	0	16
Phenylene	2	1173.0	63.7	40.4	52.4
Double bond	12	15.0	14.3	83.5	2.2
		**III**			
-F	2	102.0	493.9	6544.3	18.0
=CH-	10	255.0	38	0	13.5
$-CH_2-$	9	234.6	0	0	16.1
>C<	3	-214.2	0	0	-19.2
-OH	1	76.5	1225.0	6060.0	10.0
-CO-	1	105	600.0	9500.0	10.8
-Cl	1	397.8	1477.2	4706.0	26.0
-S-	2	815.9	196.0	297.5	12.0
=C<	7	56.7	2.00	0	5.5
-N=	3	380	100	250	5.0
>N-	5	30	150.0	750.0	-9
Ring closure 5 or more atoms	4	142.8	0	0	16
Phenylene	2	1173.0	63.7	40.4	52.4
Double bond	12	15.0	14.3	83.5	2.2

Table S3. Experimental (x_{exp}) and correlated (x_{cal}) mole fractions of compounds studied (amorphous state) solubility in the solvents studied at different temperatures and pressure p = 0.1 MPa.

T/K	x_{exp}	Modified Apelblat equation		van't Hoff equation	
		x_{cal}	[a]100RD	x_{cal}	100RD
		**I**			
		[b]Buffer pH 2.0			
293.15	$3.3040\cdot10^{-5}$	$3.3041\cdot10^{-5}$	-0.0016	$3.2958\cdot10^{-5}$	0.2490
298.15	$3.5732\cdot10^{-5}$	$3.5701\cdot10^{-5}$	0.0812	$3.5770\cdot10^{-5}$	-0.1117
303.15	$3.8570\cdot10^{-5}$	$3.8591\cdot10^{-5}$	-0.0549	$3.8717\cdot10^{-5}$	-0.3822
308.15	$4.1749\cdot10^{-5}$	$4.1729\cdot10^{-5}$	0.0510	$4.1800\cdot10^{-5}$	-0.1195
313.15	$4.5144\cdot10^{-5}$	$4.5134\cdot10^{-5}$	0.0139	$4.5018\cdot10^{-5}$	0.2709
		[c]Buffer pH 7.4			
293.15	$2.0541\cdot10^{-6}$	$2.0571\cdot10^{-6}$	-0.1443	$2.0596\cdot10^{-6}$	-0.2687
298.15	$2.2653\cdot10^{-6}$	$2.2581\cdot10^{-6}$	0.3187	$2.2563\cdot10^{-6}$	0.3991
303.15	$2.4636\cdot10^{-6}$	$2.4678\cdot10^{-6}$	-0.1689	$2.4643\cdot10^{-6}$	-0.0264
308.15	$2.6820\cdot10^{-6}$	$2.6856\cdot10^{-6}$	-0.1347	$2.6837\cdot10^{-6}$	-0.0643
313.15	$2.9135\cdot10^{-6}$	$2.9111\cdot10^{-6}$	0.0827	$2.9148\cdot10^{-6}$	-0.0443
		1-Octanol			
293.15	$1.1199\cdot10^{-3}$	$1.1199\cdot10^{-3}$	0.0057	$1.1174\cdot10^{-3}$	0.2292
298.15	$1.1981\cdot10^{-3}$	$1.2015\cdot10^{-3}$	-0.1285	$1.2023\cdot10^{-3}$	-0.1926
303.15	$1.2903\cdot10^{-3}$	$1.2886\cdot10^{-3}$	0.1103	$1.2905\cdot10^{-3}$	-0.0408
308.15	$1.3835\cdot10^{-3}$	$1.3813\cdot10^{-3}$	-0.0970	$1.3820\cdot10^{-3}$	-0.1474
313.15	$1.4834\cdot10^{-3}$	$1.4801\cdot10^{-3}$	-0.0092	$1.4768\cdot10^{-3}$	0.2166
		**II**			
		Buffer pH 2.0[a]			
293.15	$1.9402\cdot10^{-5}$	$1.9362\cdot10^{-5}$	0.1958	$1.9383\cdot10^{-5}$	0.0895
298.15	$2.2272\cdot10^{-5}$	$2.2392\cdot10^{-5}$	-0.5471	$2.2374\cdot10^{-5}$	-0.466
303.15	$2.5822\cdot10^{-5}$	$2.5740\cdot10^{-5}$	0.3105	$2.5705\cdot10^{-5}$	0.4471
308.15	$2.9451\cdot10^{-5}$	$2.9419\cdot10^{-5}$	0.1034	$2.9399\cdot10^{-5}$	0.1743
313.15	$3.3404\cdot10^{-5}$	$3.3443\cdot10^{-5}$	-0.1278	$3.3480\cdot10^{-5}$	-0.2376
		Buffer pH 7.4[b]			
293.15	$5.4794\cdot10^{-6}$	$5.4776\cdot10^{-6}$	0.0327	$5.4811\cdot10^{-6}$	-0.0301
298.15	$5.8122\cdot10^{-6}$	$5.8117\cdot10^{-6}$	0.0093	$5.8129\cdot10^{-6}$	-0.0129
303.15	$6.1623\cdot10^{-6}$	$6.1524\cdot10^{-6}$	0.1607	$6.1530\cdot10^{-6}$	0.1509
308.15	$6.4929\cdot10^{-6}$	$6.4994\cdot10^{-6}$	-0.0998	$6.5009\cdot10^{-6}$	-0.1238
313.15	$6.8581\cdot10^{-6}$	$6.8522\cdot10^{-6}$	0.0864	$6.8565\cdot10^{-6}$	0.0234
		1-Octanol			
293.15	$8.5358\cdot10^{-4}$	$8.5503\cdot10^{-4}$	-0.1679	$8.5505\cdot10^{-4}$	-0.1703
298.15	$9.0027\cdot10^{-4}$	$8.9701\cdot10^{-4}$	0.3655	$8.9678\cdot10^{-4}$	0.3908
303.15	$9.3813\cdot10^{-4}$	$9.9339\cdot10^{-4}$	-0.1371	$9.3908\cdot10^{-4}$	-0.1036
308.15	$9.8001\cdot10^{-4}$	$9.8212\cdot10^{-4}$	-0.2166	$9.8189\cdot10^{-4}$	-0.1933
313.15	$10.2644\cdot10^{-4}$	$10.2520\cdot10^{-4}$	0.1249	$10.2520\cdot10^{-4}$	0.1206
		**III**			
		Buffer pH 2.0[a]			
293.15	$1.1474\cdot10^{-5}$	$1.1471\cdot10^{-5}$	-0.0057	$1.1503\cdot10^{-5}$	-0.289
298.15	$1.2247\cdot10^{-5}$	$1.2242\cdot10^{-5}$	0.0622	$1.2225\cdot10^{-5}$	0.2079
303.15	$1.3013\cdot10^{-5}$	$1.3001\cdot10^{-5}$	0.0692	$1.2965\cdot10^{-5}$	0.3438
308.15	$1.3731\cdot10^{-5}$	$1.3742\cdot10^{-5}$	-0.0855	$1.3725\cdot10^{-5}$	0.0387
313.15	$1.4468\cdot10^{-5}$	$1.4460\cdot10^{-5}$	0.0681	$1.4502\cdot10^{-5}$	-0.2216
		Buffer pH 7.4[b]			

293.15	$1.0651\cdot10^{-6}$	$1.0639\cdot10^{-6}$	0.1126	$1.0603\cdot10^{-6}$	0.4462
298.15	$1.2262\cdot10^{-6}$	$1.2290\cdot10^{-6}$	-0.2310	$1.2312\cdot10^{-6}$	-0.4108
303.15	$1.4201\cdot10^{-6}$	$1.4179\cdot10^{-6}$	0.1558	$1.4226\cdot10^{-6}$	-0.1788
308.15	$1.6349\cdot10^{-6}$	$1.6336\cdot10^{-6}$	0.0808	$1.6361\cdot10^{-6}$	-0.0738
313.15	$1.8788\cdot10^{-6}$	$1.8796\cdot10^{-6}$	-0.0421	$1.8732\cdot10^{-6}$	0.2969
			1-Octanol		
293.15	$1.7767\cdot10^{-3}$	$1.7753\cdot10^{-3}$	0.0802	$1.7736\cdot10^{-3}$	0.1733
298.15	$1.9566\cdot10^{-3}$	$1.9588\cdot10^{-3}$	-0.1167	$1.9603\cdot10^{-3}$	-0.192
303.15	$2.1577\cdot10^{-3}$	$2.1568\cdot10^{-3}$	0.0421	$2.1595\cdot10^{-3}$	-0.084
308.15	$2.3729\cdot10^{-3}$	$2.3699\cdot10^{-3}$	0.1258	$2.3715\cdot10^{-3}$	0.059
313.15	$2.5979\cdot10^{-3}$	$2.5990\cdot10^{-3}$	-0.0415	$2.5965\cdot10^{-3}$	0.0539

[a]*RD* is the relative deviation: $RD = (x_{exp} - x_{cal})/x_{exp}$

[b]Composition of aqueous buffer pH 2.0: KCl (6.57 g in 1 L) and 0.1 mol/dm^3 hydrochloric acid (119.0 mL in 1 L)

[c]Composition of aqueous buffer pH 7.4: KH_2PO_4 (9.1 g in 1 L) and $Na_2HPO_4\cdot12H_2O$ (23.6 g in 1 L)

Standard uncertainities: $u(T)$ = 0.15 K and $u(p)$ = 3 kPa.

Relative standard uncertainties for solubility: $u_r(x)$ = 0.045 for buffer solutions and $u_r(x)$ = 0.04 for 1-octanol.

Table S4. Parameters of modified Apelblat and van't Hoff equations for of compounds studied (amorphous state) in the selected solvents.

Solvents	*A*	*B*	*C*	*RMSD*	100*RAD*
			**I**		
			Modified Apelblat equation		
Buffer pH 2.0	-77.11	-1802.3	10.67	$1.89\cdot10^{-8}$	0.04
Buffer pH 7.4	25.42	-3086.4	-4.93	$4.41\cdot10^{-9}$	0.14
1-Octanol	-48.82	813.3	6.91	$1.11\cdot10^{-6}$	0.07
			van't Hoff equation		
Buffer pH 2.0	-5.44	-1431.3	-	$9.75\cdot10^{-8}$	0.23
Buffer pH 7.4	-7.66	-1594.0	-	$4.84\cdot10^{-9}$	0.16
1-Octanol	-2.43	-1279.9	-	$2.31\cdot10^{-6}$	0.17
			**II**		
			Modified Apelblat equation		
Buffer pH 2.0	27.96	-3873.5	-4.51	$7.14\cdot10^{-8}$	0.26
Buffer pH 7.4	-2.05	-1323.5	-0.98	$5.98\cdot10^{-9}$	0.08
1-Octanol	0.32	-1038.0	-0.68	$2.03\cdot10^{-6}$	0.08
			van't Hoff equation		
Buffer pH 2.0	-2.29	-2508.7	-	$3.65\cdot10^{-8}$	0.28
Buffer pH 7.4	-8.61	-1027.7	-	$5.60\cdot10^{-9}$	0.07
1-Octanol	-4.22	-833.0	-	$2.03\cdot10^{-5}$	0.19
			**III**		
			Modified Apelblat equation		
Buffer pH 2.0	61.53	-4188.7	-10.32	$9.71\cdot10^{-9}$	0.06
Buffer pH 7.4	-87.71	-1126.9	12.34	$1.86\cdot10^{-9}$	0.12
1-Octanol	-27.56	522.4	4.05	$1.90\cdot10^{-6}$	0.07
			van't Hoff equation		
Buffer pH 2.0	-7.74	-1063.4	-	$3.10\cdot10^{-8}$	0.22
Buffer pH 7.4	-4.85	-2612.0	-	$4.17\cdot10^{-9}$	0.28
1-Octanol	-0.37	-1749.5	-	$2.48\cdot10^{-6}$	0.11

Table S5. Coefficients of empirical equation $\ln x = A - B/(1/T - 1/T_{hm})$.

Compound	A	B	R
		Buffer pH 2.0	
**I**	-10.16 ± 0.01	1431± 18	0.9997
**II**	-10.57 ± 0.01	2509± 23	0.9999
**III**	-11.26 ± 0.01	1063± 18	0.9996
		Buffer pH 7.4	
**I**	-12.92 ± 0.01	1594 ± 16	0.9998
**II**	-12.00 ± 0.01	1028 ± 6	0.9999
**III**	-13.47 ± 0.01	2612 ± 23	0.9999
		1-Octanol	
**I**	-6.66 ± 0.01	1280 ± 13	0.9998
**II**	-6.97 ± 0.01	833 ± 17	0.9994
**III**	-6.14 ± 0.01	1749 ± 9	0.9998

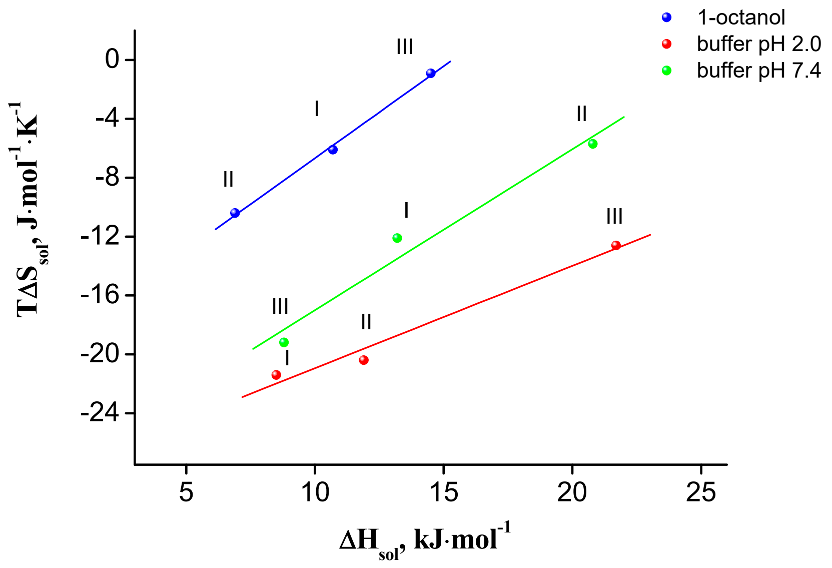

Figure S1. Relationship of the enthalpy and entropic terms of the dissolution Gibbs energy for hybrid compounds I - III in selected solvents.

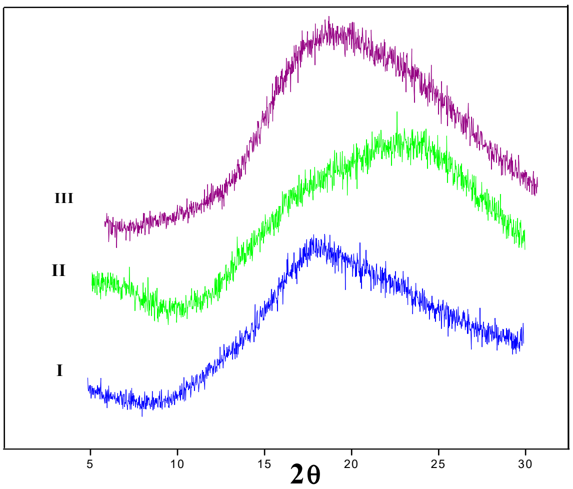

Figure S2. PXRD patterns of amorphous compounds I - III.

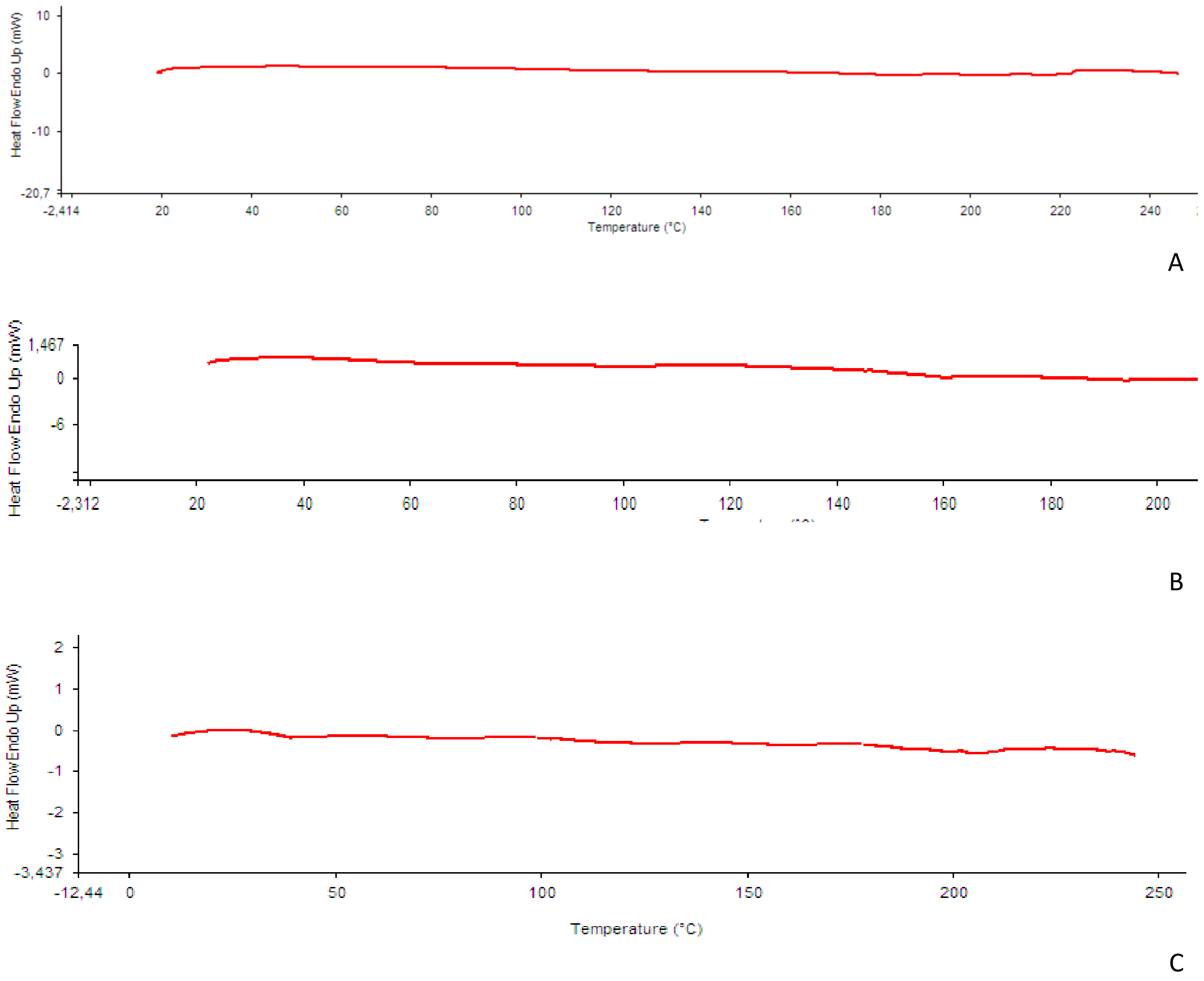

Figure S3. DSC curves of compounds I (A), II (B) and III (C).

Table S6. Density of the investigated solvents at different temperatures and pressure p = 0.1 MPa [a].

Solvent	ρ/g·cm^{-3}				
	293.15 K	298.15 K	303.15 K	308.15 K	313.15 K
Buffer pH 2.0[b]	1.0035	1.0023	1.0008	0.9992	0.9973
Buffer pH 7.4[c]	1.0060	1.0048	1.0033	1.0016	0.9998
1-Octanol	0.8251	0.8217	0.8183	0.8148	0.8114

[a]Density data for all solvents were taken from Ref. [S. Blokhina, A. Sharapova, M. Ol'khovich, G. Perlovich, A thermodynamic study of sublimation, dissolution and distribution processes of anti-inflammatory drug Clonixin, J. Chem. Thermodyn. 132 (2019) 281–288.]

[b]Composition of aqueous buffer pH 2.0: KCl (6.57 g in 1 l) and 0.1 mol/dm^3 hydrochloric acid (119.0 ml in 1 l);

[c]Composition of aqueous buffer pH 7.4: KH_2PO4 (9.1 g in 1 l) and $Na_2HPO_4 \cdot 12H_2O$ (23.6 g in 1 l);

Standard uncertainities: *u(m)*=0.01 mg, $u(T)$ = 0.15 K, $u(p)$ = 3 kPa and $u(\rho)$= 0.002 g·cm^{-3}.
